# Correlation analysis between EGFR gene mutation status, ALK positivity and demographic data, tumor biomarkers, radiological and pathological features in patients with lung adenocarcinoma

**DOI:** 10.3389/fonc.2025.1627019

**Published:** 2025-11-25

**Authors:** Xue Fei Zhang, Xu Zhang, Liang Zhao, Zhi Long Zhao

**Affiliations:** 1 Department of Thoracic Surgery, Affiliated Zhongshan Hospital of Dalian University, Dalian, Liaoning, China; 2 Department of Thoracic Surgery, The Second Hospital of Dalian Medical University, Dalian, Liaoning, China; 3 School of Software Technology, Dalian University of Technology, Dalian, Liaoning, China

**Keywords:** lung adenocarcinomas, EGFR gene mutation, ALK positivity, demographic data, tumor biomarkers, imaging, pathology

## Abstract

**Objective:**

This study aimed to explore the relationship between EGFR mutations, ALK positivity, and demographic, tumor, radiological, and pathological characteristics in lung adenocarcinoma patients.

**Methods:**

This study included 626 patients with early-stage lung adenocarcinoma who underwent surgical resection between October 2017 and December 2023.EGFR and ALK mutations were analyzed postoperatively. Clinical, pathological, and imaging features such as gender, age, smoking status, and tumor characteristics were assessed. Patients were categorized based on their mutation status, and comparisons were made regarding their clinical and imaging features.

**Results:**

Results indicated that EGFR-positive patients were predominantly female, younger, and had a higher frequency of non-smokers compared to the wild-type (WT) group. EGFR mutations, particularly the exon 19 deletions and L858R mutations, were more common in patients with moderate differentiation and lepidic or acinar predominant histological subtypes. CT imaging revealed that EGFR-positive tumors were smaller in size and had fewer solid components compared to WT tumors. Additionally, certain CT features such as the spicule sign and air bronchogram were significantly associated with EGFR mutations. For ALK mutations, the analysis showed that patients with ALK-positive tumors had distinct radiological features, including a higher occurrence in the lower lobes and fewer ground glass nodules compared to the WT group.

**Conclusions:**

The study concluded that specific radiological and pathological characteristics, along with EGFR and ALK mutation statuses, could be used to guide the treatment and diagnosis of lung adenocarcinoma.

## Introduction

Lung cancer is the leading cause of cancer-related death globally (1), with 733,300 diagnoses and 610,200 deaths in China in 2015 (2, 3). Lung adenocarcinoma is the most common type (4). In recent years, molecular-targeted therapies have transformed treatment, offering better control and less toxicity than traditional chemotherapy (5). Epidermal growth factor receptor (EGFR) mutations and anaplastic lymphoma kinase (ALK) rearrangements are the most common druggable targets in lung adenocarcinoma. EGFR mutations affect about 20% of patients, particularly in females, non-smokers, and Asians, with exon 19 deletions and exon 21 mutations being the most common (6, 7). ALK rearrangements occur in about 5% of non-small cell lung cancer (NSCLC) cases, especially in younger, light, or non-smokers, and are commonly found in adenocarcinomas (8–10).

Clinical trials (11) have shown that patients with EGFR mutations and ALK rearrangements treated with targeted therapies experience longer progression-free survival and higher response rates compared to chemotherapy. EGFR mutations includes three types (point mutation, multinucleotide in-frame deletion,and in-frame insertion) which have been documented in exon 18 through 21, highlighting that deletion mutation in exon 19 (45%) and point mutation in exon 21 (40–45%) are two most common mutations, accounting for about 90% EGFR mutations in lung adenocarcinoma (12).

The IASLC/ATS/ERS classification system for lung adenocarcinoma was proposed in 2011 (13),Though several groups had analyzed association between the IASLC/ATS/ERS classification scheme and survival (14–17), but the relationship between gene mutations and this new classification remains unclear, highlighting the need to understand correlations between gene mutations and pathological subtypes.

CT imaging plays a key role in diagnosing and assessing response in NSCLC (1, 18–25). Some studies (18–23) suggest CT features such as small lesion size and the presence of air bronchograms may be associated with EGFR mutations, while ALK mutations are linked to larger, solid masses (1, 24, 25).

However, the relationship between these mutations and the clinicopathological,imaging features features of lung adenocarcinoma has not been fully elucidated. Additionally, obtaining tissue for gene detection is difficult in advanced-stage or surgically ineligible patients, and biopsies often yield false negatives due to tumor heterogeneity and low cell counts. This has led to growing interest in identifying clinical, pathological, and radiological features associated with EGFR and ALK mutations.

## Materials and methods

### Patient selection

This retrospective study was approved by our institutional review board, which waived the need for informed consent. From October 2017 to December 2023, we identified 626 early-stage lung adenocarcinoma patients who underwent EGFR mutation and ALK rearrangement retesting after surgery at the Second Hospital of Dalian Medical University.

Inclusion criteria included pathologically confirmed lung adenocarcinoma by surgical resection, available EGFR and ALK mutation results, clinical data (age, sex, smoking history, hypertension, diabetes, tumor biomarkers), and CT scans performed within a month prior to surgery. Exclusion criteria included missing CT scans, preoperative treatments (Neoadjuvant chemotherapy, radiotherapy, targeted therapy and immunotherapy), incomplete data, severe comorbidities, or a history of lung cancer.

### Histologic evaluation and molecular analysis

Histological specimens were formalin-fixed and stained with hematoxylin-eosin. Two experienced pathologists reviewed the samples and recorded the subtype and stage according to the 8th edition TNM system and IASLC/ATS/ERS classification. EGFR mutations (exons 18-21) and ALK mutations were analyzed using next-generation sequencing (NGS). The detection is based on the Illumina sequencing platform and employs the target region probe capture technology. It examines at least 9 genes that are highly relevant to personalized treatment of non-small cell lung cancer. These genes must include EGFR, ALK, ERBB2 (HER2), BRAF, KRAS, MET, RET, ROS1, and NTRK1/2/3.ALK positivity was further confirmed by FISH testing.

### CT imaging data

**Figure 1:**
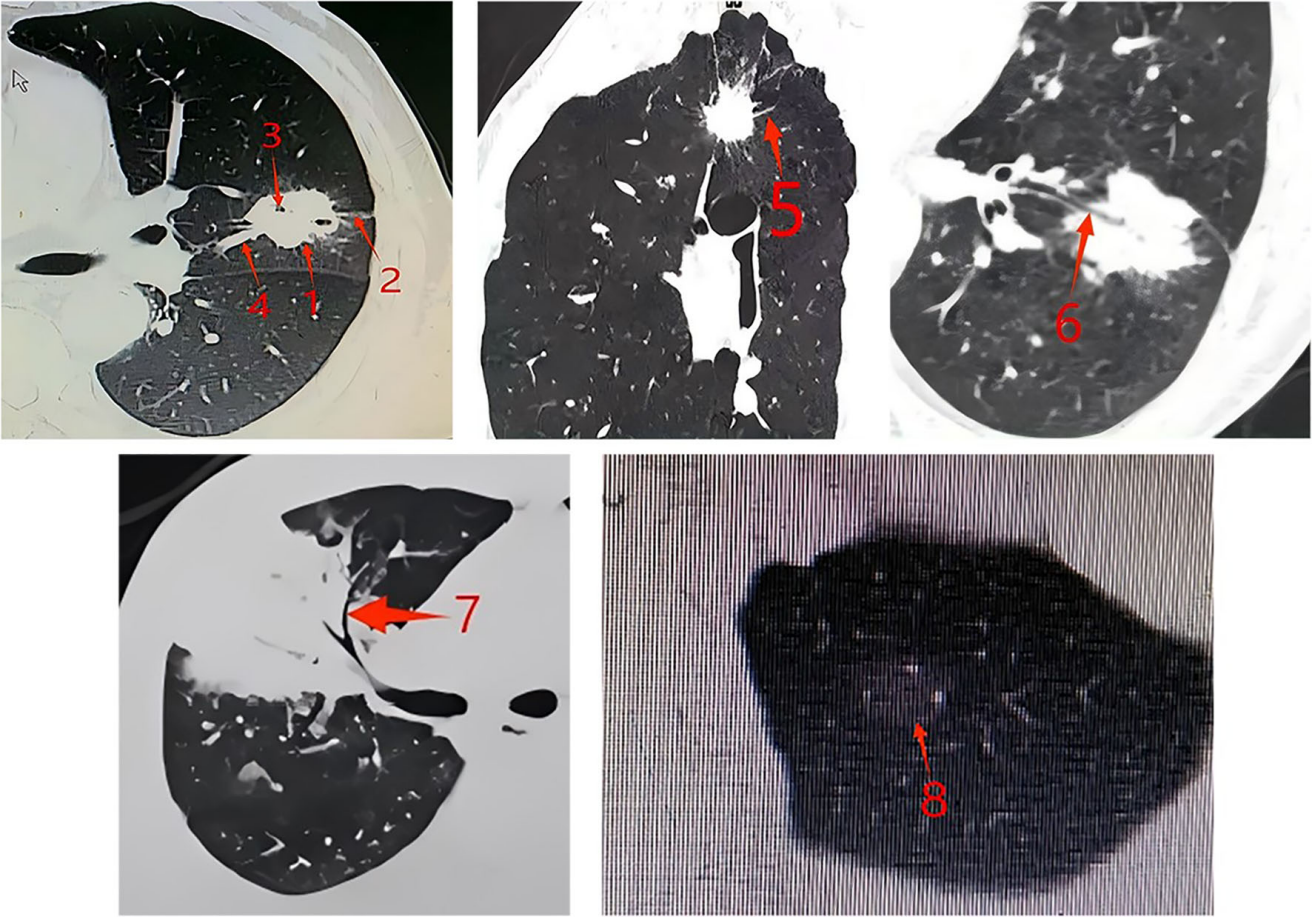
CT features of tumors. Arrow 1: Lobulation sign: The surface of the nodule is uneven, with multiple arc-shaped depressions visible on it, resembling the combined appearance of multiple nodules. Arrow 2: Pleural indentation sign: The distal pleura of the tumor is pulled by the tumor, forming a linear high-density shadow. The corresponding concave area shows a trumpet-shaped change. Arrow 3: Vacuole sign: Round, transparent shadows with a diameter of less than 5mm (mostly 1-2mm) can be seen within the lesion, either singly or in multiple clusters, and they can be located anywhere within the lesion. Arrow 4: Vessel convergence sign: Around the tumor, there is one or more blood vessels that converge towards the tumor. It can also be manifested as the blood vessels stopping or being completely surrounded and destroyed when they reach the edge of the tumor. Arrow 5: Spicule sign: The tumor margin extends outward, the base is slightly thick and gradually becomes thinner as it spreads outward, presenting a straight and forceful linear shadow. Arrow 6: Bronchial cutoff sign: The bronchus is interrupted at the edge of the lesion or as it enters the lesion. The terminal end of the lumen may be straight, blunt, or conical narrowed. The bronchial wall is locally thickened and asymmetric, gradually thinning towards the center of the lesion. Arrow 7: Air bronchogram: There are long, branched or tubular low-density shadows within the lesion, which may be accompanied by blood vessels. When perpendicular to the scanning plane, they can appear as continuous circular low-density shadows on the upper and lower layers. Arrow 8: Ground-glass nodules: The tumor nodules have a relatively low density in part or the entire area, presenting as ground-glass opacity, without obscuring the pulmonary vascular patterns, and the lesion boundaries are clear.

High-resolution CT scans (1 mm slice thickness) were reviewed by two chest subspecialists who were blind to the genetic classification. Tumor features assessed included size, location, margins (**Figure 1**), and density (pure or mixed ground-glass nodules, subsolid, or solid).

### Statistical analysis

Statistical analyses were conducted using SPSS version 21.0. An independent-sample Student’s t-test compared continuous variables, and chi-square tests compared categorical variables. Multivariate regression analysis was performed after addressing multicollinearity. A prediction tool for EGFR and ALK mutations was developed using principal component analysis, and receiver operating characteristic (ROC) curves were generated to calculate the area under the curve (AUC). Furthermore, univariate and multivariate subgroup analyses were conducted for EGFR exon 19 and exon 21 mutation sites.

## Results

### Patient characteristics and EGFR, ALK mutation status

**Figure 2:**
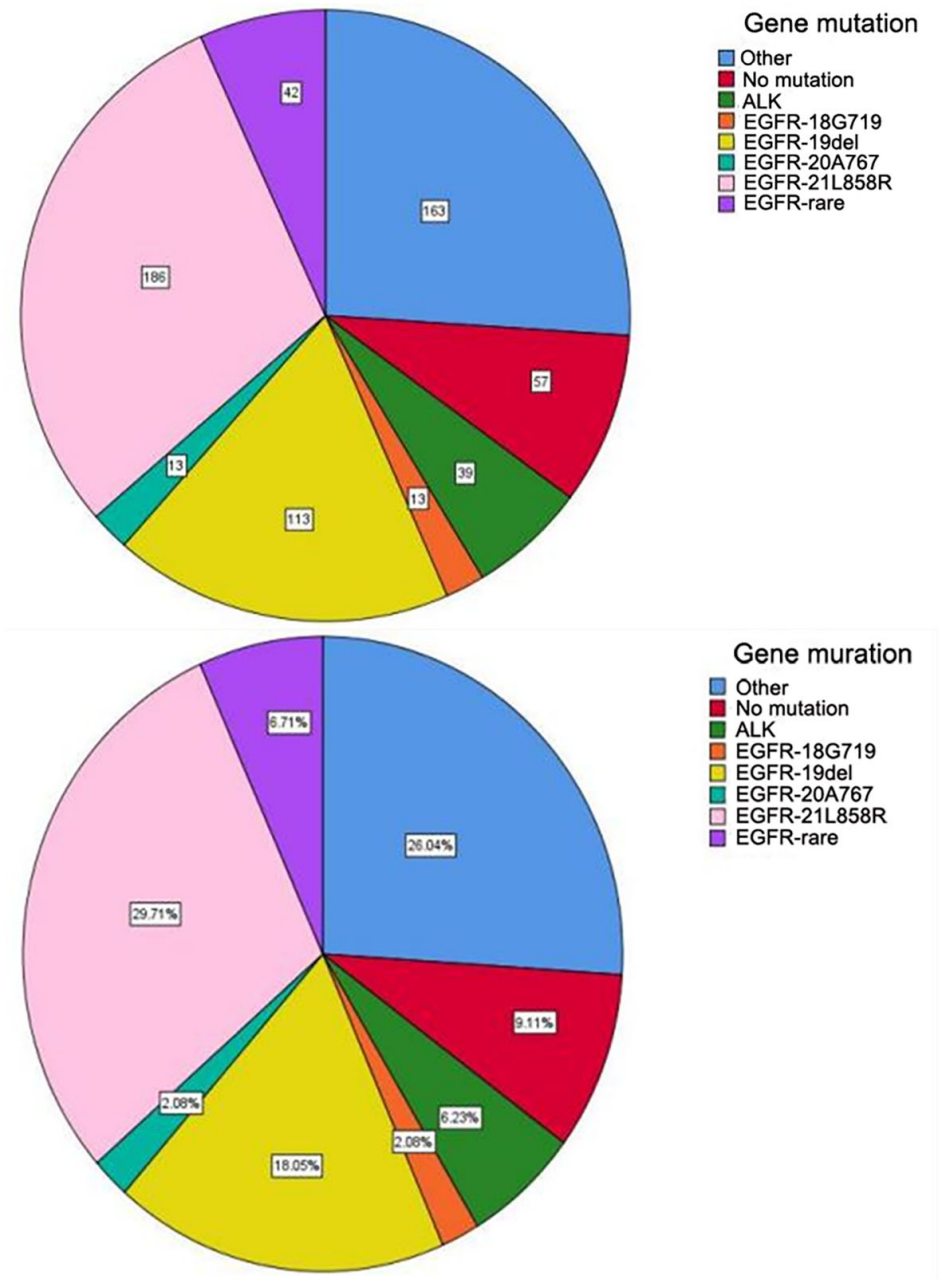
Distribution of gene mutation sites.

A total of 626 patients underwent surgery between October 2017 and December 2023, with EGFR mutation and ALK rearrangement retesting conducted post-surgery. The cohort consisted of 234 men (37.4%) and 392 women (62.6%), with a median age of 60.8 years (range 24–80). Of the patients, 452 (72.2%) were non-smokers, with 163 male and 11 female smokers. All cases were invasive lung adenocarcinomas. Among the 626 patients, 367 had EGFR mutations and 39 had EML4-ALK fusion mutations, EGFR mutations included 113 (30.8%) exon 19 deletions, 186 (50.7%) exon 21 mutations, and 68 cases (18.5%) of rare mutations (**Figure 2**).

### Correlations of EGFR mutations and ALK status with clinical, pathological and CT features

The clinical characteristics of the patients are summarized in **Table 1**. EGFR-positive patients had significantly lower mean CYFRA21–1 levels (2.358 vs. 2.796 ng/ml; P = 0.003). A higher proportion of women (66%) and non-smokers (65%) were found in the EGFR-positive group compared to the EGFR-negative group (46% and 41%, respectively; P = 0.000 for both). There were few ALK-positive patients, and no significant differences in clinical features were observed for this group.

**Table 1:** Univariate analysis of the effects of EGFR gene mutation and ALK gene mutation on clinical features of invasive lung adenocarcinoma.

Characteristics	No.	EGFR mutation status		X^2^/t	*P*	ALK mutation status		X^2^/t	*P*
		Positive (n=367)	Negative (n=259)			Positive (n=39)	Negative(n=587)		
Gender				23.965	0.000			0.291	0.590
Male	234	108	126			13	221		
Female	392	259	133			26	366		
Median age		60.89	60.68	-0.246	0.806	58.21	60.97	1.606	0.109
Age				1.074	0.300			2.103	0.147
≥60	374	213	161			19	355		
<60	252	154	98			20	232		
Smoking history				29.552	0.000			1.099	0.294
Positive	174	72	102			4.6% 8	166		
Negative	452	295	157			6.9% 31	421		
Hypertension				0.093	0.761			0.557	0.456
Positive	194	112	82			10	184		
Negative	432	255	177			29	403		
diabetes				0.019	0.890			0.138	0.710
Positive	76	44	32			4	72		
Negative	550	323	227			35	515		
CHD				0.166	0.684			1.302	0.254
Positive	19	12	7			0	19		
Negative	607	355	252			39	568		
Mean CEA (ng/ml)		2.757	3.282	0.975	0.330	1.865	3.048	1.081	0.280
Mean NSE (ng/ml)		14.127	14.220	0.277	0.782	13.656	14.199	0.790	0.430
Mean CYFRA21-1 (ng/ml)		2.358	2.796	2.996	0.003	2.538	2.540	0.006	0.995

CHD, coronary heart disease

The pathological features of surgically resected cases are shown in **Table 2**. EGFR-positive cases were more frequently lepidic and acinar (69% vs. 51% and 68% vs. 50%, respectively; P < 0.05), and less frequently solid and mucinous (37% vs. 60% and 11% vs. 62%, respectively; P < 0.05). EGFR-positive patients were more likely to be moderately differentiated (P = 0.000) and less likely to be poorly differentiated (P = 0.000). No significant pathological differences were found between ALK-positive and wild-type (WT) patients.

**Table 2:** Univariate analysis of the effects of EGFR gene mutation and ALK gene mutation on pathological features of invasive lung adenocarcinoma.

Characteristics	No.	EGFR mutation status		X^2^/t	*P*	ALK mutation status		X^2^/t	*P*
		Positive (n=367)	Negative (n=259)			Positive (n=39)	Negative (n=587)		
Differentiation				50.520	0.000			1.835	0.400
Well	151	80	71	2.615	0.106	6	145	1.735	0.188
moderate	394	266	128	34.612	0.000	28	366	1.398	0.237
Poor	81	21	60	41.016	0.000	5	76	0.001	0.982
Lepidic				19.438	0.000			2.463	0.117
Positive	268	68.7% 184	84			12	256		
Negative	358	51% 183	175			27	331		
Acinar				22.104	0.000			1.111	0.292
Positive	302	68% 206	96			22	280		
Negative	324	49.7% 161	163			17	307		
Papillary				0.009	0.925			0.065	0.799
Positive	38	22	16			2	36		
Negative	588	345	243			37	551		
Micropapillary				1.468	0.226			0.951	0.329
Positive	14	6	8			0	14		
Negative	612	361	251			39	573		
Solid				8.731	0.003			2.303	0.129
Positive	43	37% 16	27			5	38		
Negative	583	60.2% 351	232			34	549		
Mucinous				37.066	0.000			0.838	0.360
Positive	37	11% 4	33			1	36		
Negative	589	62% 363	226			38	551		
lymphatic metastasis				0.860	0.354			0.049	0.825
Positive	71	38	33			4	67		
Negative	555	329	226			35	520		

The CT features of surgically resected cases are shown in **Table 3**. EGFR mutations were significantly associated with nodule location (P = 0.013), particularly in the upper lobe of the left lung (68% vs. 56%; P = 0.011) and middle lobe of the right lung (48% vs. 60%; P = 0.035). EGFR-positive tumors had smaller solid components (1.073 vs. 1.380 cm; P = 0.003), with a higher mutation rate in ground glass nodules with 25-50% solid components (P = 0.002) and a lower rate in those with 75-100% solid components (P = 0.000) or pure solid nodules (P = 0.001). CT features such as the spicule sign (P = 0.001) and air bronchogram (P = 0.034) were more common in EGFR mutations. For ALK mutations, the mutation rate was higher in the left lower lobe (11% vs. 5%; P = 0.031), with less frequent co-occurrence of pure ground glass nodules (2.7% vs. 7.3%; P = 0.035) and vessel convergence signs (1.8% vs. 7.2%; P = 0.030).

**Table 3:** Univariate analysis of the effects of EGFR gene mutation and ALK gene mutation on radiological features of invasive lung adenocarcinoma.

Characteristics	No.	EGFR mutation status		X^2^/t	*P*	ALK mutation status		X^2^/t	*P*
		Positive (n=367)	Negative (n=259)			Positive (n=39)	Negative (n=587)		
Location of nodule				12.655	0.013			5.525	0.238
upper lobe of right lung	205	109	96	3.740	0.053	11	194	0.390	0.532
middle lobe of right lung	87	42	45	4.463	0.035	6	81	0.077	0.782
inferior lobe of right lung	104	65	39	0.772	0.380	6	98	0.045	0.831
upper lobe of left lung	130	89	41	6.543	0.011	5	125	1.596	0.206
inferior lobe of left lung	100	62	38	0.558	0.455	11	89	4.635	0.031
Mean diameter		1.990	2.070	0.878	0.380	2.254	2.008	-1.315	0.189
Diameter				0.034	0.854			0.499	0.480
≥2	271	160	111			19	252		
<2	355	207	148			20	335		
Maximum diameter of solid component		1.073	1.380	2.958	0.003	1.531	1.178	-1.662	0.097
Pure GGO				0.160	0.689			4.450	0.035
Positive	152	87	65			4	148		
Negative	474	280	194			35	439		
Solid component ratio 0-25%				0.148	0.700			2.894	0.089
Positive	206	123	83			8	198		
Negative	420	244	176			31	389		
Solid component ratio 25-50%				9.929	0.002			0.026	0.872
Positive	138	70%97	41			9	129		
Negative	488	55%270	218			30	458		
Solid component ratio 50-75%				0.639	0.424			0.163	0.686
Positive	83	52	31			6	77		
Negative	543	315	228			33	510		
Solid component ratio 75-100%				17.513	0.000			1.635	0.201
Positive	214	47%101	113			17	197		
Negative	412	64%266	146			22	390		
pure solid				11.781	0.001			0.101	0.750
Positive	163	47%77	86			11	152		
Negative	463	63%290	173			28	435		
lobulation sign				0.000	0.997			0.001	0.971
Positive	191	112	79			12	179		
Negative	435	255	180			27	408		
Spicule sign				11.440	0.001			0.421	0.516
Positive	415	263	152			24	391		
Negative	211	49%104	107			15	196		
vacuole sign				0.603	0.437			1.350	0.245
Positive	214	130	84			10	204		
Negative	412	237	175			29	383		
Pleural indentation sign				1.742	0.187			0.013	0.910
Positive	396	240	156			25	371		
Negative	230	127	103			14	216		
vessel convergence sign				0.337	0.561			4.695	0.030
Positive	113	69	44			2	111		
Negative	513	298	215			37	476		
Air bronchogram				4.484	0.034			0.164	0.685
Positive	145	74	71			8	137		
Negative	481	293	188			31	450		
Bronchial cutoff sign				0.812	0.367			2.079	0.149
Positive	56	36	20			1	55		
Negative	570	331	239			38	532		

### Differences in clinical, pathologica and CT features between 19 deletion and L858R EGFR mutations

**Table 4** shows clinical differences between EGFR exon 19 deletions and L858R mutations. Both mutations were more common in females and non-smokers compared to WT EGFR (P < 0.05). EGFR exon 19 deletions occurred more frequently in patients under 60 years of age (58.38 vs. 62.22 years; P = 0.002) and had lower mean CYFRA21–1 levels compared to WT EGFR (2.217 vs. 2.796 ng/ml; P = 0.012). Additionally, EGFR exon 19 deletions were more common in younger patients compared to L858R mutations.

**Table 4:** Univariate analysis of the effects of EGFR 19, 21 mutation on clinical features of invasive lung adenocarcinoma.

Characteristics	EGFR, n			*P*	*P1*	*p2*
	Wild type (n=259)	Exon 19 deletion (n=113)	L858R (21) (n=186)	Wild vs 19	Wild vs 21	19 vs 21
Gender				0.002	0.000	0.447
male	126	35	50			
female	133	78	136			
Median age	60.68	58.38	62.22	0.057	0.111	0.002
age				0.006	0.612	0.004
≥60	161	53	120			
<60	98	60	66			
Smoking history				0.000	0.000	0.864
Positive	102	23	33			
Negative	157	90	153			
Hypertension				0.181	0.710	0.118
Positive	82	28	62			
Negative	177	85	124			
diabetes				0.467	0.735	0.340
Positive	32	11	25			
Negative	227	102	161			
CHD				0.661	0.444	0.285
Positive	7	4	3			
Negative	252	109	183			
Mean CEA (ng/ml)	3.282	2.769	2.784	0.520	0.449	0.984
Mean NSE (ng/ml)	14.220	14.233	14.079	0.980	0.741	0.728
Mean CYFRA21-1 (ng/ml)	2.796	2.217	2.411	0.012	0.046	0.224

CHD, coronary heart disease

**Table 5** shows the pathological features of EGFR 19 deletion and L858R mutations. Both mutations were more commonly associated with moderate differentiation (P = 0.000) and high frequencies of lepidic and acinar subtypes, while being rare in mucinous subtypes. EGFR 21 mutations were also rare in solid pathological subtypes (16% vs. 41%; P = 0.002).

**Table 5:** Univariate analysis of the effects of EGFR 19, 21 mutation on pathological features of invasive lung adenocarcinoma.

Characteristics	EGFR, n			*P*	*P1*	*p2*
	Wild type (n=259)	Exon 19 deletion (n=113)	L858R (21) (n=186)	Wild vs 19	Wild vs 21	19 vs 21
Differentiation				0.000	0.000	0.083
Well	71	18	43	0.017	0.306	0.135
moderate	128	86	137	0.000	0.000	0.637
Poor	60	9	6	0.001	0.000	0.069
Lepidic				0.001	0.000	0.941
Positive	84	57	93			
Negative	175	56	93			
Acinar				0.000	0.000	0.320
Positive	96	68	101			
Negative	163	45	85			
Papillary				0.745	0.257	0.203
Positive	16	8	7			
Negative	243	105	179			
Micropapillary				0.820	0.158	0.302
Positive	8	3	2			
Negative	251	110	184			
Solid				0.193	0.002	0.134
Positive	27	7	5			
Negative	232	106	181			
Mucinous				0.000	0.000	−
Positive	33	0	0			
Negative	226	113	186			
lymphatic metastasis				0.739	0.317	0.615
Positive	33	13	18			
Negative	226	100	168			

**Table 6** shows the CT features of EGFR 19 deletion and L858R mutations. Compared to WT EGFR, EGFR 21 mutations were more frequent in the upper lobe of the left lung (P = 0.038) and rare in the middle lobe of the right lung. EGFR L858R mutations had smaller solid components (1.030 vs. 1.380 cm; P = 0.007), with higher mutation rates in ground glass nodules with 25-50% solid components (P = 0.001). Both EGFR 19 and L858R mutations were rare in ground glass nodules with 75-100% solid components or pure solid nodules. EGFR 19 mutations were more likely to occur in ground glass nodules with 50-75% solid components. The CT characteristics that significantly differed between EGFR mutations and WT EGFR were the spicule sign (P = 0.011, P = 0.003). EGFR 21 mutations were more likely to show a bronchial cutoff sign (P = 0.034).

**Table 6:** Univariate analysis of the effects of EGFR 19, 21 mutation on radiological features of invasive lung adenocarcinoma.

Characteristics	EGFR,n			*P*	*P1*	*p2*
	Wild type (n=259)	Exon 19 deletion (n=113)	L858R (21) (n=186)	Wild vs 19	Wild vs 21	19 vs 21
Location of nodule				0.301	0.028	0.199
upper lobe of right lung	96	33	60	0.143	0.294	0.580
middle lobe of right lung	45	18	17	0.733	0.013	0.077
inferior lobe of right lung	39	23	29	0.207	0.877	0.292
upper lobe of left lung	41	25	44	0.144	0.038	0.760
inferior lobe of left lung	38	14	36	0.559	0.191	0.118
Mean diameter	2.070	2.002	2.032	0.623	0.743	0.791
Diameter				0.700	0.205	0.167
≥2	111	46	91			
<2	148	67	95			
Maximum diameter of solid component	1.380	1.150	1.030	0.136	0.007	0.384
Pure GGO				0.322	0.499	0.141
Positive	65	23	52			
Negative	194	90	134			
Solid component ratio 0-25%				0.708	0.268	0.216
Positive	83	34	69			
Negative	176	79	117			
Solid component ratio25-50%				0.098	0.001	0.254
Positive	41	26	54			
Negative	218	87	132			
Solid component ratio50-75%				0.057	0.254	0.006
Positive	31	22	16			
Negative	228	91	170			
Solid component ratio75-100%				0.009	0.000	0.591
Positive	113	33	49			
Negative	146	80	137			
pure solid				0.049	0.002	0.522
Positive	86	26	37			
Negative	173	87	149			
Lobulation sign				0.550	0.929	0.525
Positive	79	38	56			
Negative	180	75	130			
Spicule sign				0.011	0.003	0.998
Positive	152	82	135			
Negative	107	31	51			
vacuole sign				0.168	0.307	0.638
Positive	84	45	69			
Negative	175	68	117			
Pleural indentation sign				0.262	0.205	0.966
Positive	156	75	123			
Negative	103	38	63			
vessel convergence sign				0.437	0.288	0.899
Positive	44	23	39			
Negative	215	90	147			
Air bronchogram				0.150	0.068	0.923
Positive	71	23	37			
Negative	188	90	149			
Bronchial cutoff sign				0.401	0.071	0.034
Positive	20	6	24			
Negative	239	107	162			

### Multivariable analyses of prognostic factors for EGFR, EGFR19 and 21 mutation and ALK mutation and receiver operating characteristic curve analysis

Multivariate analysis (**Table 7**) identified several independent predictors of EGFR mutations: gender, moderate differentiation, lepidic, acinar, and mucinous subtypes, middle lobe of the right lung, spicule sign, and air bronchogram (P < 0.05). For EGFR exon 19 mutations, independent predictors included age, degree of differentiation, lepidic, acinar subtypes, 75-100% solid component ratio, and spicule sign (P < 0.05). For EGFR exon 21 mutations, gender, moderate differentiation, lepidic, acinar subtypes, and spicule sign were significant (P < 0.05). Pure GGO and vessel convergence sign were independent predictors of ALK gene fusion (P < 0.05).

**Table 7:** Independent predictors of each subtype of EGFR mutation and ALK gene fusion.

Characteristics	EGFR		EGFR 19		EGFR 21		ALK	
	OR (95%CI)	*P*	OR (95%CI)	*P*	OR (95%CI)	*P*	OR (95%CI)	*P*
Smoking history	1.564 (0.862-2.836)	0.141	1.769 (0.770-4.062)	0.179	1.586 (0.771-3.261)	0.210		
Age			0.557 (0.323-0.961)	0.035				
Gender	0.554 (0.318-0.968)	0.038	0.662 (0.306-1.430)	0.293	0.474 (0.243-0.925)	0.029		
Mean CYFRA21-1	0.920 (0.811-1.045)	0.198	0.819 (0.656-1.022)	0.077	0.931 (0.802-1.079)	0.341		
Poor	1.741 (0.657-4.614)	0.265	4.988 (1.548-16.075)	0.007	1.545 (0.412-5.792)	0.519		
moderately	2.535 (1.469-4.374)	0.001	4.389 (2.026-9.505)	0.000	2.407 (1.254-4.621)	0.008		
Well			0.259 (0.074-0.909)	0.035				
Lepidic	3.860 (2.324-6.411)	0.000	3.991 (2.009-7.926)	0.000	3.661 (1.938-6.917)	0.000		
Acinar	2.710 (1.643-4.470)	0.000	2.421 (1.240-4.726)	0.010	2.422 (1.274-4.603)	0.007		
Solid	0.899 (0.346-2.335)	0.827			0.636 (0.176-2.300)	0.49		
Pure GGO							0.308 (0.107-0.888)	0.029
Mucinous	0.171 (0.049-0.593)	0.005	0.000	0.998	0.000	0.998		
Upper lobe of left lung	1.402 (0.872-2.257)	0.164			1.235 (0.707-2.157)	0.459		
Inferior lobe of left lung							1.995 (0.948-4.196)	0.069
Middle lobe of right lung	0.556 (0.316-0.980)	0.042			0.522 (0.256-1.066)	0.074		
Solid component ratio25-50%	1.338 (0.793-2.257)	0.275			1.483 (0.803-2.741)	0.208		
Solid component ratio75-100%	0.483 (0.212-1.100)	0.083	0.327 (0.119-0.901)	0.031	0.606 (0.216-1.699)	0.341		
Pure solid	1.205 (0.554-2.619)	0.638	1.682 (0.578-4.894)	0.340	1.044 (0.415-2.625)	0.927		
Spicule sign	2.329 (1.480-3.666)	0.000	2.636 (1.441-4.821)	0.002	2.120 (1.242-3.620)	0.006		
Air bronchogram	0.534 (0.335-0.852)	0.009						
Vessel convergence sign							0.194 (0.046-0.822)	0.026
Diameter of solid component	1.013 (0.797-1.288)	0.914			1.011 (0.760-1.346)	0.938		

### ROC

**Figure 3:**
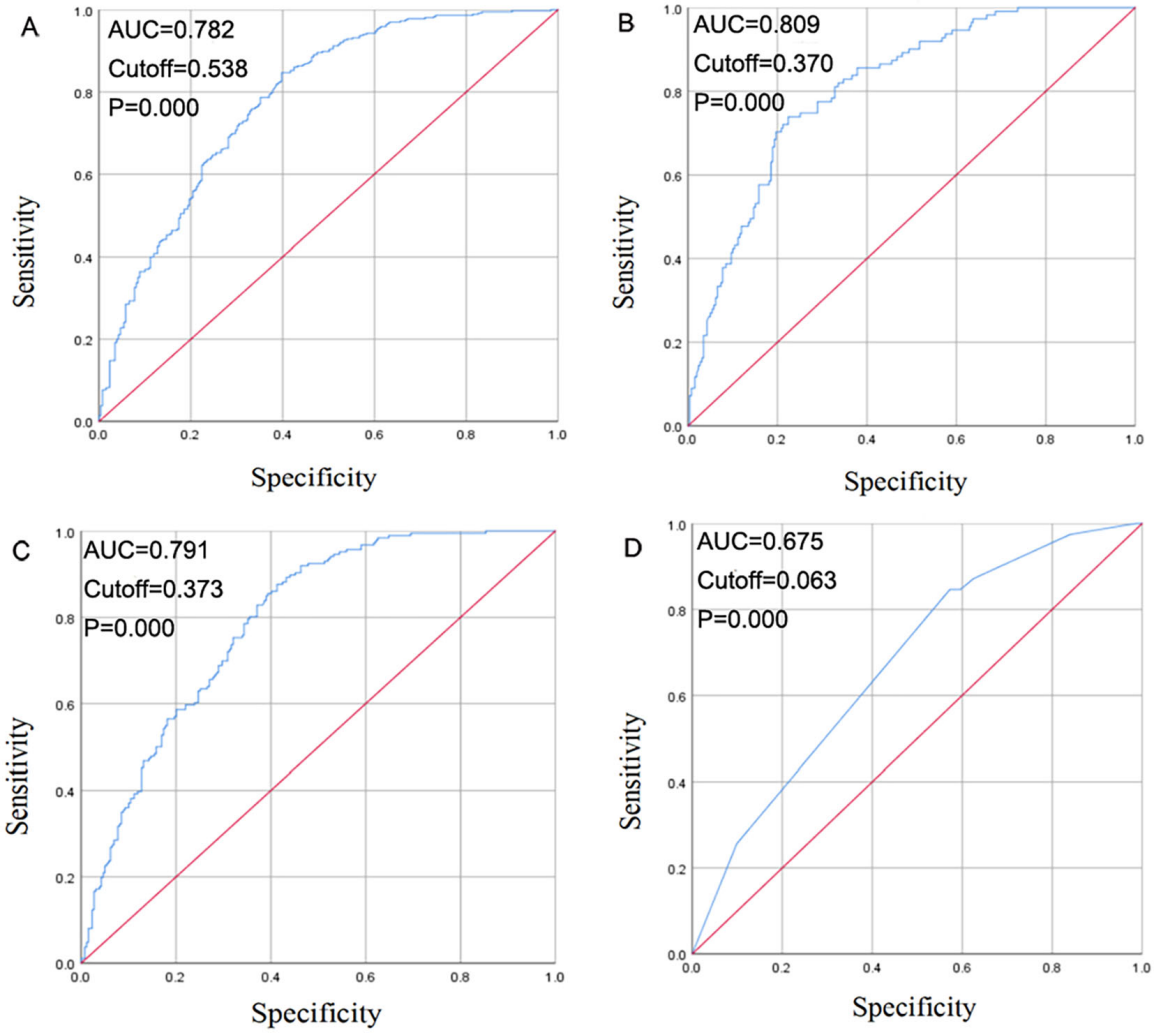
Receiver operating curve (ROC) curve for EGFR mutation **(A)**, EGFR 19 mutation **(B)**, EGFR 21 mutation **(C)**, ALK positivity **(D)** prediction.

To validate the logistic regression model for predicting EGFR, EGFR19, EGFR21, and ALK mutation status, ROC curves were generated using the regression equations. The ROC for EGFR showed an AUC of 0.782 (P = 0.000, 95% CI: 0.745-0.819), for EGFR19 an AUC of 0.809 (P = 0.000, 95% CI: 0.764-0.855), for EGFR21 an AUC of 0.791 (P = 0.000, 95% CI: 0.750-0.832), and for ALK an AUC of 0.675 (P = 0.000, 95% CI: 0.595-0.755) (**Figure 3**).

## Discussion

EGFR and ALK mutations are recognized as critical drivers in the development of NSCLC, especially lung adenocarcinoma, and serve as important targets for clinical therapy. These mutations play a pivotal role in diagnosis and treatment planning (26). This study aims to explore the correlation between EGFR mutation status, ALK positivity, and various demographic, radiological, and pathological features of lung adenocarcinoma.

This study included 626 lung adenocarcinoma patients, with EGFR mutations found in 58.63% (367/626) and ALK gene rearrangements in 6.23% (39/626) of cases. These rates were consistent with previous studies (27). Univariate and multivariate analyses were conducted, and ROC curves were plotted to evaluate the diagnostic value of clinical, radiological, and pathological features in predicting EGFR and ALK mutation statuses. Subgroup analysis was performed for different EGFR mutation sites, focusing on exon 19 and exon 21 mutations, to assess the impact of specific mutation types.

### Clinical characteristics of EGFR and ALK mutations

The study reaffirmed that EGFR mutations are more prevalent in non-smoking women, which aligns with prior studies by Paez et al. (28) and Lynch et al. (29). The PIONEER study (6) also demonstrated a high EGFR mutation rate (51.4%) in Asian women and non-smokers with lung adenocarcinoma. In this study, smoking history did not emerge as an independent predictor of EGFR mutations in multivariate analysis, suggesting that smoking may not play a direct role in EGFR mutation status in this cohort, likely due to sample bias from the single-center study.

The results of this study showed that there was no correlation between the mean age or the 60-year age stratification and EGFR mutation status, which is consistent with previous literature (30, 31). Although some studies have suggested that diabetes may influence tumor biology and EGFR signaling pathways, thus participating in tumor development (32–34), no association between diabetes and EGFR mutation rate was found in this study. Hypertension and coronary heart disease also did not show a significant correlation with EGFR mutation.

Regarding tumor markers, although some studies have reported that elevated CEA levels are associated with a higher EGFR mutation rate (35–37), its independence as a risk factor remains controversial. NSE levels are not significantly correlated with EGFR mutation (38), but it may serve as a predictor of EGFR-TKI treatment response and a prognostic indicator for patients with EGFR mutations (39). In this study, the mean CYFRA21–1 level in the mutation group was lower than in the WT group (2.358 vs. 2.796 ng/ml, P = 0.003), but multivariate regression analysis did not identify it as an independent predictor of EGFR mutation. This is consistent with the retrospective study by Takeuchi et al. (40).

In terms of EGFR mutation subtypes, this study found that EGFR exon 19 and 21 mutations were more common in women and patients without a smoking history, which is in line with the conclusions of Cao et al. (41). However, after multivariate analysis, smoking was not an independent risk factor. Female gender was an independent predictor for EGFR exon 21 mutation, and EGFR exon 19 mutations were more common in patients under 60 years of age compared to those with wild-type EGFR, which was an independent predictor after multivariate analysis. The CYFRA21–1 level was lower in patients with EGFR exon 19 mutations but was not an independent predictor after regression analysis. Patients with EGFR exon 19 mutations were younger than those with exon 21 mutations (58.38 vs 62.22 years, P = 0.002).

Compared to EGFR gene mutations, ALK rearrangement is more likely to occur in younger, non-smoking or light-smoking lung adenocarcinoma patients (42). In this study, it was also found that the average age of patients with ALK mutations was younger, and the mutation rate was significantly higher in those under 60 years old. The mutation rate in patients without a smoking history was higher than in those with a smoking history. However, due to the small number of ALK fusion cases, no statistical difference was found. The research on the proportion of male and female ALK fusion mutation-positive patients is still controversial (43, 44). In this study, the ALK mutation rate was slightly higher in female patients, but no significant statistical difference was observed. There were no significant differences in tumor markers, hypertension, diabetes, or coronary heart disease.

### Differences in pathological characteristics between EGFR and ALK mutations

Regarding tumor differentiation, this study found that EGFR mutations were more common in moderately differentiated adenocarcinomas and rare in poorly differentiated tumors, which slightly differs from previous research suggesting a lower mutation rate in poorly differentiated cases (45). In general, better tumor differentiation correlates with a better prognosis, supporting that EGFR mutation detection aligns with tumor differentiation in determining lung cancer prognosis.

Histopathological subtypes of lung adenocarcinoma show varying sensitivities to molecular-targeted drugs. Studies have indicated that certain subtypes, such as papillary and lepidic, have higher EGFR mutation rates, although the exact relationship remains unclear (46, 47). In this study, the mutation rate was higher in lepidic and acinar dominant subtypes, consistent with previous reports (21, 48–50). Previous studies (51) also showed that lepidic as the main subtype manifested as GGO on CT.The more ground glass components of GGO, the higher the EGFR mutation rate, which was consistent with the above results.Conversely, solid and mucinous subtypes had a lower EGFR mutation rate, with mucinous subtypes showing rare mutations, aligning with Inamura et al.’s findings (52).

Further analysis of EGFR mutations at exon 19/21 sites showed higher mutation rates in moderately differentiated adenocarcinomas, particularly in lepidic and acinar subtypes, and low mutation rates in mucinous subtypes. Solid subtypes also had a low incidence of EGFR21 mutations, though multivariate analysis did not identify this as an independent predictor.

Regarding ALK mutations, Wang et al. reported an association with solid dominant subtypes, which was also observed in this study, though the difference was not statistically significant (53). Several studies suggest ALK mutations are linked to lymph node metastasis, indicating poor prognosis (54–56). However, this study found no significant correlation between ALK mutations and lymph node metastasis, likely due to the inclusion of early-stage tumors and no significant differences in tumor differentiation or histological subtypes.

### Differences in Imaging Characteristics Between EGFR and ALK Mutation Populations

Regarding lesion location, previous studies have shown no correlation between lesion location and EGFR mutation status (23, 57). However, some studies suggest that EGFR mutations are more common in the upper lung lobes, possibly due to a complex interaction between genetic and environmental factors (58). In this study, univariate analysis found a higher EGFR mutation rate in tumors located in the upper lobe of the left lung and a lower rate in the middle lobe of the right lung. Multivariate regression analysis identified the middle lobe of the right lung as an independent risk factor for EGFR mutations, indicating the need for further research to determine whether lobe distribution in peripheral small lung adenocarcinomas can predict EGFR mutations.

In terms of CT features, early lung adenocarcinomas often present as pure ground glass opacity (pGGO), partial solid nodules, or solid nodules. Previous studies have suggested a relationship between EGFR mutations and solid components of GGO (59, 60), although findings are inconsistent. Most studies report that EGFR mutations correlate with a higher proportion of GGO (61, 62), suggesting that GGO could predict EGFR mutations. However, other studies (63, 64) found that EGFR mutations were more common in lesions with more than 50% solid components. This study showed that EGFR mutation patients had smaller maximum solid component diameters, with a higher mutation rate in tumors with 25-50% solid components and a lower rate in tumors with 75-100% solid components. In pure solid nodules, the mutation rate was also low, supporting a positive correlation between EGFR mutations and GGO components. The presence of solid components in GGO may indicate increased tumor aggression and pathological grade (60).

CT features like the air bronchogram, spicule sign, and tumor size have been linked to EGFR mutations. Some studies suggest a positive correlation between air bronchogram and EGFR mutation (58, 65), while others report a negative correlation (66). In this study, EGFR mutations were positively correlated with the spicule sign and negatively correlated with the air bronchogram, with no significant correlation found with other features. Multivariate analysis identified spicule sign and air bronchogram as independent predictors of EGFR mutations.

Further analysis of EGFR mutation subtypes, specifically exon 19/21 mutations, found that EGFR21 mutations were more common in the upper lobe of the left lung and rare in the middle lobe of the right lung, consistent with Tengeng et al.’s study (58). However, multivariate regression showed no correlation between lesion location and EGFR mutation. Solid components of GGO in EGFR21 patients were smaller than in EGFR19 patients, and EGFR21 mutations were more frequent in patients with 25-50% solid components, consistent with Lee et al.’s findings (18). The mutation rate increased with the proportion of GGO. Both EGFR19 and EGFR21 mutations had lower mutation rates in pure solid nodules or GGO with 75-100% solid content and higher rates with spicule signs. There was no significant difference in the air bronchogram between the two mutation types.

In terms of ALK mutations, previous studies have shown that ALK gene fusion is associated with larger tumor volumes and higher solid component proportions (55). This study found the same trend, but no statistical significance due to the small number of ALK fusion cases. ALK mutations were more common in the left inferior lobe, but this was not an independent predictor. Studies have shown that ALK mutations are associated with larger tumor diameters and higher solid component proportions, which was confirmed in this study, although no significant statistical differences were observed due to the small sample size. Pure GGO nodules had a very low ALK mutation rate (P<0.05), with mutation rates increasing as the solid component proportion increased.

Other studies (67–71) have shown that ALK-positive tumors are less likely to exhibit cavity signs and air bronchograms, and are associated with larger tumor size and a higher proportion of solid components. In this study, ALK mutations were less common in patients with vessel convergence signs, with no statistical differences found in other imaging features. This may be related to the fact that the lung cancer patients in this study were early-stage and had fewer lymph node or distant metastases.

### Multi-factor analysis of EGFR and ALK mutations

Multivariate analysis identified several independent predictors for EGFR mutations: female gender, moderate differentiation, lepidic and acinar subtypes, mucinous subtype, middle lobe of the right lung, spicule sign, and air bronchogram. Subgroup analysis revealed that age under 60, degree of differentiation, lepidic and acinar subtypes, 75-100% GGO solid component, and spicule sign were independent predictors for EGFR19 mutation. For EGFR21 mutation, female gender, moderate differentiation, lepidic and acinar subtypes, and spicule sign were independent predictors. Pure GGO and vessel convergence sign were independent predictors for ALK mutation.

An ROC curve analysis showed the diagnostic value of clinical, pathological, and HRCT models for predicting EGFR, EGFR19, EGFR21, and ALK mutations. The AUC values were 0.782 for EGFR, 0.809 for EGFR19, 0.791 for EGFR21, and 0.675 for ALK, indicating that the combined radiomic and clinical feature models can effectively predict gene mutation status.

Limitations: (1) The study was a single-center, retrospective analysis with 100% Asian patients, which may introduce selection bias. (2) The low ALK fusion rate resulted in fewer positive cases, and differences from previous studies may be due to the early-stage tumors. Further research with a broader patient population and extended follow-up is needed to assess the relationship between gene mutations, clinicopathological features, and prognosis. (3) Only early-stage lung adenocarcinomas were included, so it remains unclear whether the findings apply to advanced stages.

## Conclusion

This study found a correlation between EGFR mutation status, ALK positivity, and demographic, tumor, radiological, and pathological features in lung adenocarcinoma patients. Although CT imaging and pathological features cannot replace the role of molecular testing, to a certain extent, they can assist in predicting the genetic mutation status and further determine which patients may be suitable for receiving EGFR-TKIs or ALK inhibitors treatment.

## Data Availability

The original contributions presented in the study are included in the article/supplementary material. Further inquiries can be directed to the corresponding authors.
